# Emotional Nuance Enhances Verbatim Retention of Written Materials

**DOI:** 10.3389/fpsyg.2021.519729

**Published:** 2021-06-14

**Authors:** Yoonji Kim, Diana Van Lancker Sidtis, John J. Sidtis

**Affiliations:** ^1^Department of Communicative Sciences and Disorders, New York University, New York, NY, United States; ^2^Brain and Behavior Laboratory, Nathan Kline Institute for Psychiatric Research, New York, NY, United States; ^3^Department of Psychiatry, New York University Langone School of Medicine, New York, NY, United States

**Keywords:** emotion, episodic memory, constructionist approach, recognition, verbatim memory

## Abstract

Recent studies have demonstrated that details of verbal material are retained in memory. Further, converging evidence points to a memory-enhancing effect of emotion such that memory for emotional events is stronger than memory for neutral events. Building upon this work, it appears likely that verbatim sentence forms will be remembered better when tinged with emotional nuance. Most previous studies have focused on single words. The current study examines the role of emotional nuance in the verbatim retention of longer sentences in written material. In this study, participants silently read transcriptions of spontaneous narratives, half of which had been delivered within a context of emotional expression and the other half with neutral expression. Transcripts were taken from selected narratives that received the highest, most extreme ratings, neutral or emotional. Participants identified written excerpts in a yes/no recognition test. Results revealed that participants’ verbatim memory was significantly greater for excerpts from emotionally nuanced narratives than from neutral narratives. It is concluded that the narratives, pre-rated as emotional or neutral, drove this effect of emotion on verbatim retention. These findings expand a growing body of evidence for a role of emotion in memory, and lend support to episodic theories of language and the constructionist account.

## Introduction

The role of memory in language has been the major focus of an extensive body of empirical research over the past half century. It has interested scholars across a wide range of disciplines ranging from linguists and psychologists to cognitive neuroscientists. Originating from a classic study by Sachs (1967), the propositional theory in psychology holds that the verbatim forms of sentences (exact wording) are no longer retained after comprehension takes place, with only the gist (semantic content) of verbal materials being remembered (Bartlett, 1932; Anderson and Bower, 1973). This idea is consistent with the tenets of Chomsky (1957, 1965, 1981) generative grammar, which presupposes that language users infer a generalization from individual items. In opposition to these accounts, the episodic theory in psychology (Tulving, 1983) proposes that people retain the verbatim forms of sentences in long-term memory in addition to the gist of those sentences. Consistent with the episodic account of speech perception (Hintzman, 1986; Palmeri et al., 1993; Nygaard et al., 1994; Goldinger, 1996), the constructionist approach (Goldberg, 2006) in linguistics proposes that our knowledge of language includes both item-specific information and generalizations. The current study is in support of episodic and constructionist account demonstrating the existence of verbatim memory for written language.

A burgeoning literature has demonstrated that emotion enhances memory (Cahill and McGaugh, 1995; Dolcos et al., 2017). It has been shown that emotional experiences are powerfully ingrained in memory, creating vivid memories that persist longer than neutral experiences. A recent study found that emotionally expressed voices are more durably stored in long term memory than neutral voices even after a brief exposure (Kim et al., 2019). Although there is a substantial body of work attesting to the memory-enhancing effect of emotion, little is known about how the nuances of emotion influence the retention of verbal information in the written modality.

A primary goal of this study was to examine the role of emotional nuance in encoding verbatim information of written language in memory. Operationally, emotional nuance was defined as narratives or excerpts produced with high emotional expression and high engagement ratings. We assessed recognition memory of emotional and neutral written excerpts taken from transcribed spontaneous narratives. Based on the episodic and constructionist accounts, we pursued the hypothesis that episodic details of verbal materials would be specifically encoded in memory and further strengthened when laced with emotional nuance. That is, emotional nuances would boost the verbatim retention of written text, resulting in higher recognition memory of verbatim text in emotional contexts than in matched neutral contexts.

### Verbatim Memory for Language

The existence of verbatim memory for language has generated considerable support from the constructionist approach to language. Also known as construction-based grammar, the constructionist approach posits that language is composed of a network of constructions, learned pairings of form and function/meaning (Goldberg, 1995, 2006, 2013). While rejecting the idea of grammatical structure devoid of meaning, the constructionist approach ties well with the proposals of the usage-based model of language by arguing that structure emerges from use (Bybee, 1985, 2013; Langacker, 1988; Kemmer and Barlow, 2000; Tomasello, 2003). Based on this idea, the usage-based model of language emphasizes that the gist as well as the verbatim information in a sentence coexist in memory. Empirical support for this hypothesis comes from Gurevich et al. (2010). In Gurevich et al. (2010), listeners viewed a series of pictures taken from a children’s book while listening to a recorded narration. They were then presented with the written test clauses and for each clause, they indicated whether the clause had been previously heard in the story. The results revealed that listeners successfully distinguished between the text in verbatim form and in a paraphrased form, which lends support to the existence of verbatim memory. A similar perspective arises from studies of speech perception. The proposal that we retain significant amounts of verbatim language is in line with the central tenet of episodic theory wherein episodic details (phonetic and vocal information) of what we hear are encoded in memory (Hintzman, 1986; Goldinger, 1996; Johnson, 1997, 2006; Mitterer and Reinisch, 2017).

It is interesting to note that “surface” forms of sentences are retained especially well when the materials imply pragmatic inferences such as sarcasm (Murphy and Shapiro, 1994), politeness (Holtgraves, 1997), or highly interactional content (Keenan et al., 1977; MacWhinney et al., 1982). The study by Keenan et al. (1977) showed superior surface memory for sentences that were “high in interactional content” (p. 549). These sentences typically contain pragmatically significant information, such as sarcasm, jokes, and personal criticisms, which reflect close interaction between speaker and listener. These were contrasted with poor surface memory for sentences that are “low in interactional content,” those conveying only propositional information. These findings suggest that high interaction statements were more memorable, supposedly because of their rich pragmatic connotations. Being interpersonal in nature, pragmatic information has a tendency to invite listeners to personally engage in the context, likely increasing memorability. In contrast, low interaction statements do not provide such subjective support, and are thus less likely to be remembered. Children remembered naturalistically spoken idioms better than matched novel expressions after a single exposure, presumably due to their saliency and affective connotations (Reuterskiöld and van Lancker Sidtis, 2013).

Similarly, in the current study, it was hypothesized that emotional nuance will encourage readers to personally engage with the text, thereby enhancing the reading experience of the text for the reader. In order to elicit emotional nuance in a spontaneous speech monolog, we used transcribed narratives that were spontaneously produced by performers with emotional or neutral expression. The narratives obtained from the performers were rated in terms of emotional expressiveness and engagement to select the texts that represent extremes on both continua for the recognition task. This procedure was done to ensure that differences in overall emotional or neutral expression between the two types of narratives are easily discerned. Using these materials, we constructed two types of written narratives: ones rich in emotional nuance, referred to as “emotional narratives” and ones devoid of nuance, referred to as “neutral narratives.” In the selected narratives, strong emotional expressiveness or neutral, indifferent expression was present throughout the respective discourse units. Unlike prior studies that examined verbatim memory for spoken language (Keenan et al., 1977; Gurevich et al., 2010), this study particularly focused on the written modality, specifically, the extent to which verbatim memory may be influenced by emotional nuance conveyed in written language. As such, select narratives as well as the test excerpts from the narratives were all presented in a written format. (See Table 1 for example of emotional and neutral narratives).

**TABLE 1:** Example of emotional and neutral narratives used in the study.

Emotional narrative:
…wonderful. And I’m kind of relaxed but I’m also kind of stressed. And he says like sit down, and so we sit down. And I’m like, I’m like antsy. I’m like okay we got to get to where we got to get to. And he’s like okay let’s let’s get to the dinner. And I’m like great. And so I get up and I’m like you know – go back to like – walking really fast or whatever. And he’s – he stands up and he’s like wait wait wait wait wait. And I’m like, what? What do you want? I have done everything that you’ve wanted. And I walk up to him and he goes down on a knee. And I don’t even know what he said. He like, I think he like mumbled something cause he was so excited. Cause he was acting so strange the entire time. He was acting so strange. He mumbled cause he was excited and I was like – and I was, I literally, I was like what. What’s happening? What’s happening? And so then I did my cry. And like anybody who knows me, I do like the ugly cry. It’s not like, I’m adorable, it’s like, it’s like I’m a goblin. A goblin cry. And so he was – it was really – but it was really great. It was really great.
Neutral narrative:
…definitely a good, a good waste of time in the sense of a party but for young girls it took a lot more time than the expert. And I remember vividly that all the girls in this moment would watch the owner of the store in amazement to see how she had done something so quickly that we had just spent an hour and a half to 2 h doing. And then of course everyone would eat cake afterwards wearing their new necklaces. And get to go home…point on we would go back to my house and do a lot of face paint. And also um eat ice cream Hoodsie cups which I’m not sure if that’s just a regional thing. But where I’m from that’s definitely a staple at all birthday parties – is a small ice cream cup with vanilla or chocolate, they come together half and half. And you eat it with a small wooden flat spoon stick. And girls always leave with…

### A Powerful Effect of Emotion on Memory

An extensive body of empirical research confirms that emotional experiences generate highly durable and vivid memories (Brown and Kulik, 1977; Heuer and Reisberg, 1990). The vast majority of studies investigating the role of emotion in language have used words as stimuli: emotional words are significantly better remembered than neutral words (Rubin and Friendly, 1986; Phelps et al., 1997; LaBar and Phelps, 1998; Colombel, 2000; Doerksen and Shimamura, 2001; Kensinger et al., 2002). Brierley et al. (2007) revealed that emotional context surrounding individual words contributes to emotional memory enhancement. In their study, neutral words embedded in emotionally arousing sentences elicited superior recognition as compared to the same words embedded in neutral sentences. The emotional memory effect has also been found in studies using slides that are accompanied by pre-recorded narratives (Cahill and McGaugh, 1995; Laney et al., 2004). For example, Laney et al. (2004) demonstrated that emotional arousal enhances memory for the gist, central and peripheral details about the event. An investigation of persons with aphasia has revealed that presenting emotional words or sentences significantly enhanced language performance in oral reading and writing to dictation (Landis et al., 1982), auditory word repetition (Ramsberger, 1986), and auditory word discrimination tasks (Reuterskiöld, 1991).

Emotional content in language has been commonly identified along the dimensions of valence and arousal (Bradley and Lang, 1994; Russell, 2003). In the first study to empirically explore the affective dimensions underlying words (Osgood et al., 1975), three principal dimensions have been proposed: valence (positive – negative), arousal (calm – aroused), and potency (weak – strong). Later, the concept of Core Affect, put forward by Russell (2003), viewed emotion as a combination of valence and arousal in bipolar dimensions. In line with Russell’s approach, Bradley and Lang (1994, 2009) also focused on only two dimensions as determining emotional connotations of words. Studies examining the valence-arousal interaction on other cognitive processing, such as attention (Jefferies et al., 2008), creativity (Tidikis et al., 2017), and reading (Citron et al., 2014), showed that the changes in two factors affect the effect of emotion in an interactive way. Although valence and arousal were not part of the study’s independent variables, a series of text analyses were undertaken in a post hoc manner, using Linguistic Inquiry and Word Count (LIWC; Pennebaker et al., 2015) and Affective Norms for English Words (ANEW; Bradley and Lang, 2010) to further explore affective connotations of words with reference to the two dimensions.

### Aims and Hypotheses of the Current Study

Based on previous work studying the impact of emotional significance on language processing, it seemed reasonable to assume that people retain episodic details of verbal materials when laced with emotional nuance. Yet existing literature does not provide an adequate understanding of the link between emotion and verbatim memory of written language beyond the lexical level. The general aim of this study was to explore the effect of emotional nuance on verbatim memory in written language. Specifically, are verbatim excerpts deriving from emotional narratives better remembered than those from neutral narratives? To this end, participants silently read transcriptions of spontaneous narratives, half of which had been produced with high emotional expressions and half with neutral, as originally spoken by performers. The text excerpts included excerpts taken from the narratives that participants had seen (target excerpts), and excerpts from narratives by the same performers that participants had not seen (foil excerpts). They subsequently completed a yes/no recognition test, in which they distinguished seen from unseen written excerpts. Within each condition, half of these test excerpts were emotional and the other half were neutral; half had been seen previously and half had not. It was anticipated that participants would recognize verbatim excerpts taken from previously seen emotionally nuanced narratives better than excerpts taken from the previously seen neutral narratives or the unseen narratives.

## Materials and Methods

The current study was approved by the Institutional Review Board (IRB) at New York University.

### Participants

Sixteen healthy native speakers of English (13 females) participated as readers in the study. Participants ranged in age from 18 to 34 years (*M* = 24.1, *SD* = 4.9). The age of one female participant was not recorded, but assumed to be in her 20’s based on her status as a Master’s student at the time of testing. All had learned English as a first language from birth. All participants were native speakers of American English except for one British and one Singaporean English speaker. All participants had normal hearing and signed informed consent form prior to the beginning of the study. One participant who failed to follow instructions was excluded, and thus the final sample consisted of fifteen participants (12 females).

*A priori* power analysis was carried out using GPower (Faul et al., 2007) to calculate the necessary sample size for an effect size of 0.8 [large effect defined by Cohen (1988)]. The program was set to the *t*-tests, to means: differences between two dependent means (matched pairs) and to the “*A priori*: Compute required sample size” power analysis. The analysis indicated that a sample of 15 would be sufficient to detect a large sized effect with an alpha = 0.05 and power = 0.80.

### Stimuli

The stimuli were transcribed narratives from video recordings of spontaneous speech produced in emotional or neutral tones by performers. These performers (28 females) were recruited from an improvisational and comedy training center to provide emotionally intoned and neutral narratives. All acquired American English as a native language from birth and ranged in age from 20 and 40 years. Each performer was randomly assigned to an emotional or a neutral presentation of the narrative and instructed to talk freely about topics of their choice for 3 min using the intended tone of voice (emotional or neutral). A list of topics that are common to adult English speakers was provided as prompts (e.g., birthday party, wedding, graduation, family, and car ride), but narrators were encouraged to suggest any topics that they were interested to talk about. They could also create fictional scenarios and use emotions of different or mixed valences (positive and negative). For the emotional condition, they were asked to tell their stories in an expressive, emotively extreme manner; for the neutral condition, they were asked to tell their stories with neutral expression, using a calm, deliberate manner of delivery. Two or three narratives were elicited by each performer, each of which lasted for 3 min, resulting in 85 narratives in total. All narratives were audio- and video-recorded and carefully transcribed verbatim, including all verbal detail for presentation in the experiment.

The obtained videotaped narratives were then submitted to five adult female raters, all of whom were native speakers of American English. The goal was to identify extremely emotional and neutral videotaped narratives, as listeners perceive them. The raters were instructed to watch each videotaped narrative (*N* = 85) and then rate the degree of (1) emotional expressiveness and (2) engagement, respectively, on two 7-point rating scales. Participant instructions for the video ratings were provided in Table 2. The first rating, emotional expressiveness, reflected the intensity of expressed emotion in narration, ranging from 1-neutral (*very little to no emotional expression*) to 7-expressive (*high emotional energy*). The second rating, engagement, was to quantify the extent to which the performer is engaging in her narrative, ranging from 1 (*not at all*) to 7 (*very engaging*). The ratings were obtained from spoken materials, instead of written transcripts. It was our intention to see if the overall emotional vs. neutral status of the spoken narratives is conveyed in written form. Our view was that each narrative, as a whole, conveyed an overall emotional or neutral orientation as a Gestalt; excerpts taken from transcripts of these narratives should carry enough of this material to influence participants.

**TABLE 2:** Participant instructions for video ratings.

Please watch the videos in the order they appear. We would like to perform two ratings on each video. The first is emotional expressiveness as heard in voice, intonation, prosody, and melody of speech in story telling. Your ratings will be placed on a continuum from “neutral” (1-very little or no emotional expression) to “expressive” (7-high emotional energy). The second is a rating of how engaging the performer is in her story telling, again on a scale where 1 = “not at all engaging” and 7 = “very engaging.” Please take care to match the header of the video with the label on the rating sheet.

We calculated the average of two ratings to select the final 24 narratives, separating the narratives into the extremes of emotional and neutral expression. Half of the narratives represent emotional extremes, whereas the other half represent neutral extremes. Table 1 presents a typical example of emotional and neutral narratives used in the study (see Supplementary Material for a complete list of narratives).

From this pool of narratives, two stimulus sets (set A and set B) were devised, each containing 12 transcribed narratives. Half of the videos in each stimulus set consisted of emotional narratives and half contained neutral narratives (see Table 3). The average length of narratives was 184 words, each approximately 1 min in duration. The performers were matched between narratives in sets A and B, with different content, in order to balance personal narrative style. The topics covered in the two sets of narratives were largely matched, centering around career choice, marriage, birthday, and family (see Supplementary Material for a list of topics covered in each narrative).

**TABLE 3:** Number of narratives and excerpts for two stimulus sets.

	**Set A**	**Set B**	**Total**
Emotional narratives	6	6	12
Neutral narratives	6	6	12
Narratives in total	12	12	24
Emotional excerpts	30	30	60
Neutral excerpts	30	30	60
Excerpts in total	60	60	120

A list of 120 test excerpts was obtained from the two stimulus sets, half of which were taken from the emotional and half taken from neutral narratives. Five excerpts were selected from each narrative (5 excerpts 24 narratives = 120 excerpts). There was thus a total of 60 (set A: 30 emotional + 30 neutral) + 60 (set B: 30 emotional + 30 neutral) = 120 excerpts presented for each participant (see Table 3). All excerpts ranged in length from 10 to 15 words and had a mean duration of 2–3 s.

The test stimuli consisted of written excerpts from transcriptions of narratives that participants have read (target excerpts), and excerpts from transcriptions of narratives by the same speakers that they have not read (foil excerpts). In two cases, proper nouns were removed from the test stimuli. The eliminated words were replaced with an ellipsis consisting of three dots, as in: “So last week I got cast in a new… show at the,” and “and I invited my friend from high school,…, with us.” Table 4 illustrates examples of emotional and neutral excerpts (see Supplementary Material for a full list of excerpts).

**TABLE 4:** Example of emotional and neutral excerpts used in the study.

Emotional excerpts:
1) …and it’s really exciting…um…you know I actually have been 2) special to me. I’ve never had a real dog. Um so 3) he’s like okay let’s let’s get to the dinner. And I’m like great. 4) the middle of nowhere, we finally see another car. and my moms like 5) And luckily I got the younger sister princess who is- she’s bored 6) I think it’s really stupid. and like people who do it are like dumb 7) so that’ll be good. I actually just recently watched a video 8) about it. Then I’m like you know I actually read this. 9) didn’t know anything about the city. you know just kind of 10) like, what? What do you want? I have done everything that you’ve
Neutral excerpts:
1) family was there. And it’s one of those days where you just feel 2) things. So they had five or so friends who were 3) a really great day. I mean, my best friends were there. 4) and it’s something that I’ve seen uh more than once on the 5) Um and that’s why I don’t go with them anymore cause I have a 6) I feel like is a thing everywhere. Um so as I 7) getting married. She um is marrying someone she’s been with for about 8) show us around. I kinda do wish that it was happening 9) hotel and put it on the cake. And then later 10) got the warm weather now. And I have been spending my days uh

#### Text Analyses

In order to more directly evaluate the status of the written material with respect to the independent variable (emotion and engagement as a characteristic of the overall transcribed narrative), detailed corpus analyses of the narratives and the excerpts were performed, using two methods: LIWC (Pennebaker et al., 2015) and ANEW (Bradley and Lang, 2010). The LIWC is an automated text analysis program, originally developed as a method for studying linguistic and psychological elements in verbal materials. Specifically, the LIWC program matches words in a text to its internal dictionary of 6,500 words to classify them into 90 categories. For each text file, the LIWC generates an output file which displays a list of selected categories, each expressed as a percentage of total word count. For the purpose of this study, the current analysis specifically focused on one LIWC category, namely “affective processes.”

The ANEW is a standardized material for quantitative analysis of emotional words in the English language. Specifically, based on the dimensional view of emotion in psychology (i.e., the semantic differential, established by Osgood et al., 1957), the ANEW describes a list of about 1,000 words that convey emotion along with normative emotional ratings (ranging from 1 to 9) of three dimensions for each word, namely valence, arousal and dominance. Words in the narratives were matched to the ANEW word list. Any inflected (e.g., verb inflections indicating a past tense *-ed*, noun inflections indicating a plural *-s*), or derived words (e.g., adjective-to-noun derivation as in *slow* and *slowness*, verb-to-noun derivation as in *write* and *writer*) whose stems are matched to the word list and contractions (e.g., *wanna*, contraction of *want to*) were considered.

As emotion words are represented by the two dimensions of valence and arousal (Osgood et al., 1957), for this study, the matched words were examined in terms of these two norms. Also, these two dimensions were used to capture the words that are not equated for valence and arousal. For example, according to the ANEW list, words such as *crazy* are arousing, but are neutral on valence scale; words such as *ugly* are negatively valenced, but are not arousing.

To this end, words high in valence (7.0–9.0, indicating positive emotion) or low (1.0–3.9, indicating negative emotion) in valence were categorized as valenced words, while words rated between these two poles (4.0–6.9) were categorized as neutral words. Similarly, words high in arousal (6.0–9.0) were considered arousing, whereas words rated lower than 6 in arousal were considered nonarousing. For the purpose of this study, “emotional words” include the words that are evaluated as either (1) valenced (positive or negative) on the valence dimension or (2) arousing on the arousal dimension.

### Procedure

All participants were tested individually in a quiet room during a single session lasting approximately 1 h. Participants were seated in a comfortable chair and stimuli were presented on paper. Before starting the experimental session, participants filled out a list of background questions pertaining to their gender, educational background, and native language.

The experiment consisted of two phases: an exposure phase and a recognition test phase. In the exposure phase, participants were instructed to silently read written stories originally narrated by female speakers. Participants were informed that they would subsequently be asked to recognize the excerpts taken out of the stories. Each participant read one of two sets of 12 transcribed narratives (set A or set B), self-paced. Accordingly, half of the participants read six emotional and six neutral narratives in set A, while the other half read six emotional and six neutral narratives in set B. The narratives were presented in a pseudo-random order so that no more than three narratives from the same category appeared sequentially. The narratives appeared in the same order in each stimulus set. The mean number of transcript words across narratives was 184 words. The total group of stories for each participant constituted between 2097 (set A) and 2,388 words (set B) depending on which set was assigned to the participant.

In the subsequent recognition test phase, the participants were told that they would be presented with written excerpts, some of which were taken from the stories that they had read and some were not. For each excerpt presented, participants indicated whether the excerpt had been previously seen during exposure. They were instructed to select “Yes” if they saw it before at encoding or “No” if they did not. The order of excerpts was randomized, with each participant receiving the same sequence.

The test phase consisted of a total of 120 trials, containing half emotional (*N* = 60) and half neutral written excerpts (*N* = 60). A total of 60 excerpts taken from the subset of narratives that participants read (A or B) were presented along with additional 60 excerpts taken from the other subset of unexposed narratives (B or A). To ensure an unbiased selection, the test excerpts were randomly chosen by the experimenter blind to the emotional/neutral distinction of the narratives. Since participants were exposed only to narratives from either set A or set B and the test excerpts were taken from both sets (sets A and B), half of the excerpts had been witnessed before (thus, the correct answer was “yes” for yes/no recognition test) and half of the excerpts were new (thus, the correct answer was “no” for yes/no recognition test) for each participant. Such a design, called a block design, was used to control for any possible effects that might arise from the salience of words or linguistic structures in narratives, which is not of primary interest in this study.

## Results

Based on signal detection theory, the dependent measures for the recognition task included recognition accuracy measured by d-prime (*d*′) and response bias measured by *c*. The formula for *d*′ was *d*′ = *z*(*hit rate*) − *z*(*false alarm rate*) and the formula for *c* was *c* = −0.5[*z*(*hit rate*) + *z*(*false alarm rate*)] (Stanislaw and Todorov, 1999). Table 5 provides the mean number of hits, false alarms, correct rejections, and misses for the emotional and neutral excerpts. All statistical analyses were performed with the SPSS software (IBM Corp., Armonk, NY, United States).

**TABLE 5:** Mean number of hits, false alarms, correct rejections, and misses for the emotional and neutral excerpts. Standard deviations are given in parentheses.

**Condition**	**Hits**	**False alarms**	**Correct rejections**	**Misses**
Emotional excerpts	21.7 (4.6)	8.1 (4.0)	21.9 (4.0)	8.3 (4.6)
Neutral excerpts	20.3 (5.0)	8.8 (4.8)	21.1 (4.7)	9.7 (5.0)

### Recognition Accuracy (*d*′)

Overall, the mean recognition accuracy (*d*′) was 1.22 (*SD* = 0.34) across participants. A one-sample *t*-test on recognition accuracy (*d*′) revealed that the mean recognition accuracy (*d*′) was significantly greater than zero, *t*(14) = 13.71, *p* < 0.001, Cohen’s *d* = 3.54. These results indicate that participants reliably recognized the differences between excerpts that they had seen verbatim (i.e., target excerpts) and excerpts originally obtained from the same speakers but with different content (i.e., foil excerpts). These results also suggest that written materials were encoded verbatim in memory and such verbatim memories were accessed on the subsequent test to endorse whether the written excerpts have been seen before.

**FIGURE 1:**
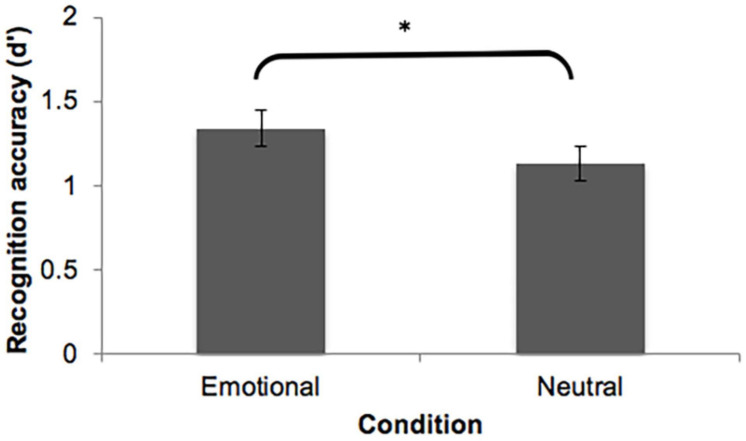
Mean recognition accuracy (*d*′) plotted as a function of mode (emotional vs. neutral excerpts), averaged across all participants. Error bars represent standard error of the mean. Significant differences are denoted by asterisks, ^∗^*p* < 0.05.

Table 6 presents mean hit rates (HR), false alarm rates (FAR), and recognition accuracy (*d*′) as a function of mode (emotional vs. neutral excerpts). In order to examine whether emotion affects the retention of verbatim representations, we conducted a paired-samples *t*-test on recognition accuracy (*d*′) for excerpts taken from emotional and neutral excerpts. Verbatim representation was significantly better recognized for excerpts taken from the emotional narratives (*M* = 1.34, *SD* = 0.36) than excerpts taken from the neutral narratives (*M* = 1.13, *SD* = 0.4), *t*(14) = 2.43, *p* = 0.029, Cohen’s *d* = 0.63. This finding confirms that participants were more likely to retain verbatim representations of excerpts from emotional narratives compared to the ones from neutral narratives. An overview of the results can be seen in Figure 1.

**TABLE 6:** Mean hit rates (HR), false alarm rates (FAR), and recognition accuracy (*d*′) for emotional and neutral excerpts.

	**Emotional excerpts**	**Neutral excerpts**
HR	0.72 (0.15)	0.68 (0.17)
FAR	0.27 (0.13)	0.29 (0.16)
*d*′	1.34 (0.36)	1.13 (0.4)

*Standard deviations are provided in parentheses.*

### Response Bias (*c*)

Overall, the mean response bias (*c*) was 0.03 (*SD* = 0.43) across participants. A one-sample *t*-test demonstrated that the overall response bias *c* was not significantly different from 0, *t*(14) = 0.31, *p* = 0.763, Cohen’s *d* = 0.08, indicating that there was neither a bias toward responding *yes* nor a bias toward responding *no*.

A paired-samples *t*-test was conducted to investigate whether a response criterion shifts depending on the mode. The results showed no significant differences in response bias between emotional (*M* = 0.01, *SD* = 0.43) and neutral excerpts (*M* = 0.06, *SD* = 0.12), *t*(14) = −0.67, *p* = 0.516, Cohen’s *d* = 0.17. That is, participants were not inclined to favor either the *yes* response or the *no* response for both emotional and neutral excerpts.

### LIWC Analysis

#### Emotional Words in Narratives

Using the LIWC data, an independent sample *t*-test revealed that there are statistically significantly higher proportions of emotional words in emotional transcribed narratives compared to neutral narratives, *t*(22) = 2.36, *p* = 0.014, Cohen’s *d* = 0.96, *M*_*emotional*_ = 6%, *SD*_*emotional*_ = 1.92% vs. *M*_*neutral*_ = 4.15%, *SD*_*neutral*_ = 1.89%. Given that each narrative averaged 184 words in length, there were, on average, about 11 emotional words (184 × 0.06 = 11) in emotional narratives, and about 7 emotional words (184 × 0.04 = 7) in neutral narratives.

#### Emotional Words in Excerpts

The emotional and neutral excerpts were analyzed using LIWC. The LIWC results showed that there were greater proportions of emotional words in emotional excerpts (6.2%) vs. neutral excerpts (3.51%). Despite the difference in proportions of emotional words in emotional excerpts vs. neutral excerpts, it is worth noting that the numerical differences between the two were fairly small. Considering that our data was expressed as a percentage, the average number of emotional words in each excerpt was less than a word (0.5 words) and many excerpts contain 0% of emotional words. Due to many data points that were zero, no statistical analyses were conducted on these data. These data reflect the experimental design that excerpts were randomly selected throughout the two sets of narratives. This result suggests that presence or absence of words classified as emotional was not the key determining factor in participants’ responses. This supports our hypothesis that the original narratives transmitted sufficient affective nuance to be discerned in excerpts taken from them.

### ANEW Analysis

#### Emotional Words in Narratives

Using the ANEW identification procedure, an independent sample *t*-test showed that there was no significant difference in overall proportion of emotional words contained in emotional vs. neutral narratives, *t*(22) = 0.882, *p* = 0.387. When the overall analysis using the ANEW list failed to reach statistical significance, careful inspection of their ratings provided a clue that emotional valence (i.e., positivity and negativity) and arousal levels differed for the independent variable. That is, the results of independent samples *t*-test indicate that there are greater proportions of negative, *t*(22) = 2.879, *p* = 0.009, and arousing words, *t*(22) = 2.539, *p* = 0.019, in the emotional narratives, than in the neutral narratives. In contrast, there was no significant difference in the proportions of positive words in emotional and neutral narratives, *t*(22) = 0.888, *p* = 0.384. The means and standard deviations of these measures are reported in Table 7.

**TABLE 7:** Mean percentages (standard deviations) for different types of ANEW words included in emotional and neutral narratives.

**Types of words**	**Mode of narratives**	***M* (*SD*)**	**Significance (*p*-value)**
Positive	Emotional	95.4 (39)	0.384
	Neutral	80.7 (41.9)	
Negative	Emotional	11.1 (8)	0.009
	Neutral	3.6 (4.4)	
Arousing	Emotional	61.7 (30.7)	0.019
	Neutral	33.9 (22.3)	

#### Emotional Words in Excerpts

As was the case with LIWC, each excerpt contained only 0–3 words that match those in the ANEW list, limiting statistical analyses. The proportion of emotional words in emotional excerpts was 7.73% (62 words / 802 words × 100 = 7.73%) and 6.74% (50 words / 742 words × 100 = 6.74%) in neutral excerpts. Overall, there was a negligible effect of positivity in emotional and neutral excerpts: emotional excerpts contained 5.36% positive words, while neutral excerpts contained 5.66%. The proportions of negative and arousing words in the two types of excerpts mirrored those in the narratives. Emotional excerpts expressed more negative and arousing words than did the neutral excerpts (negative words: 1.25% vs. 0.67%, arousing words: 4.61% vs. 2.83%). See Table 8 for the number of words that matched each ANEW category. While interesting, these data are minimal and do not allow for statistical comparisons.

**TABLE 8:** Proportions of different types of ANEW words contained in emotional and neutral excerpts.

**Types of words**	**Mode of excerpts**	**Proportion of words in excerpts**
Positive	Emotional	5.36% (43 words / 802 words)
	Neutral	5.66% (42 words / 742 words)
Negative	Emotional	1.25% (10 words / 802 words)
	Neutral	0.67% (5 words / 742 words)
Arousing	Emotional	4.61% (37 words / 802 words)
	Neutral	2.83% (21 words / 742 words)

## Discussion

Building on previous literature on the effect of language and emotion on verbal memory, the goal of the present study was to investigate the influence of emotionally nuanced verbal materials on verbatim memory. To address this, memory for emotional and neutral written excerpts, taken from written narratives seen or unseen by readers, was assessed through a recognition test. First, participants were able to successfully endorse whether or not they had seen excerpts presented to them, corroborating the existence of verbatim memory for written language originally spoken in a spontaneous manner. Second, a key finding of this study is a facilitative role for emotional nuance in verbatim retention of verbal materials; participants demonstrated better recognition memory for excerpts from transcribed emotional narratives compared to those from transcribed neutral narratives. This pattern of results establishes that emotional nuance enhances verbatim retention of written excerpts and further highlights the ability of written language to communicate emotional nuance in spontaneous discourse.

The narratives used in the experiment were originally spoken by performers with emotional or neutral expression and then selected by raters according to which narratives received the highest and lowest ratings for emotional expressiveness and engagement to ensure that distinctions between emotional and neutral texts were significantly large. According to the result of the lexical analysis, measures of emotionally laden lexical items in the excerpts and transcripts reveal small differences between emotional and neutral stimuli. It is likely that the difference between the emotional and neutral narratives lies in the overall, pervasive emotional nuance or neutral context carried in each narrative as a whole. We conclude that the web of emotional nuance created by emotional connections throughout the discourse is accountable for the impact on the participants.

Evidence of verbatim memory for language obtained in this study is consistent with the claim of current notable episodic (or exemplar) and constructionist theories that the verbatim form of text is specifically represented and maintained in memory (Kintsch and Bates, 1977; Reyna and Kiernan, 1994; Gurevich et al., 2010). Structural priming studies substantiate this idea, showing that speakers tend to produce sentence structures they have encountered previously (Bock, 1986; Szmrecsanyi, 2005; Ferreira and Bock, 2006). A recent study by Luka and Choi (2012) found that exposure to a certain sentence structure modifies speakers’ syntactic preference and such preference was pronounced even after a 3-day delay. In their study, participants were asked to read a set of sentences out loud, and then assess grammatical acceptability for three kinds of sentences on a 7-point rating scale: exactly the same, of a similar structure, and a different sentence structure. Results indicated that identical sentences and sentences with similar structure were rated as more acceptable than sentences with a different structure. This finding can be interpreted as evidence that sentence structures, regarded as surface details peripheral to sentence content or meaning, are stored robustly in memory and are persistent over time. Although the present study tested verbatim memory in written modality, our results fit well with evidence from the speech perception literature since the 1990s. It has been shown that listeners retain perceptual details of spoken language and employ this information for later processing. Such perceptual details include intonation contour, pitch, (Church and Schacter, 1994), pitch contour shape (Kimball and Cole, 2016), rate (Green et al., 1997; Bradlow et al., 1999), stress (Bellik and Roberts, 2019), and voice information (Palmeri et al., 1993; Nygaard et al., 1994; Goldinger, 1996).

A fundamental claim of the constructionist theory is that linguistic structures, combined with function, are stored as constructions and emerge from actual usage of these constructions (Goldberg, 2013). The present findings are in agreement with this usage-based constructionist view, in that constructions were encoded and recognized verbatim, validating the presence of usage-based knowledge of language in readers. The present investigation attempted to examine verbatim memory in a more ecologically valid context by introducing an emotional component. Considering that natural speech in daily life normally imbued with more or less presence of subtle nuances of attitude and emotion, we used spontaneous narratives as the basis for participants’ responses. The narratives were originally produced in extremes of either emotional and neutral expression. As the role of written materials in communicating emotions has been poorly understood, relative to the spoken materials, the current investigation, focusing on written modalities, advances our understanding of how the emotional component comes into play in encoding the verbatim wording of written discourse.

With regard to stimuli, it is worth noting that the transcribed narratives used in the current study were long enough to ensure that participants were unlikely encode or rehearse the sentences intentionally. The entire narratives averaged 2,242.5 words in length (2,097 words for set A, 2,388 words for set B) (compare this to ≈ 300 words in Gurevich et al., 2010). Also, the text excerpts taken from the narratives did not contain any salient words or proper nouns, which might serve as an inference cue for recognizing excerpts.

The distinctiveness and attention-grabbing nature of emotional stimuli has been shown to drive performance. These cognitive characteristics of emotional stimuli are closely related to each other because relatively distinctive items automatically capture attentional resources (Theeuwes, 1994; Franconeri and Simons, 2003). The distinctive attributes of emotional stimuli have received attention from researchers as a key to enhancing memory (Loftus and Burns, 1982; Christianson and Loftus, 1987; Schmidt, 1991, 2002). The idea that emotional stimuli stand out relative to neutral stimuli was investigated by Dewhurst and Parry (2000), who compared the recognition of emotional and neutral words in pure-list vs. mixed-list designs. Dewhurst and Parry (2000) reported that the emotional enhancement effect arose only when emotional and neutral words were presented together in a mixed-list design and disappeared when they were presented separately in a pure-list design. These results suggest that it was the relative distinctiveness of the emotional stimuli that boosted recognition memory (Hadley and MacKay, 2006; Schmidt and Saari, 2007). The current study, in which the emotional and neutral narratives were intermixed within a list, replicates this emotional effect, indicating that distinctive features of emotional stimuli played a role in enhancing verbatim memory.

In a similar vein, an increased attention toward emotional stimuli can also account for the preferential encoding of verbatim representations for the emotional narratives. There has been a substantial amount of study demonstrating that emotional stimuli trigger attentional capture, leading to prioritized processing (Pessoa et al., 2002, 2005; Mitchell et al., 2007; Pourtois et al., 2010; Oliveira et al., 2013). Consistent with these findings, recent neuroimaging studies have provided insights into neural mechanisms underlying selective attention to emotional information (Vuilleumier et al., 2001; Anderson et al., 2003). These studies have shown that brain circuits centering on the amygdala are selectively activated during selective attention in response to emotion-laden stimuli. It is thus plausible that that excellent verbatim retention of emotional narratives can be partially attributed to a reliance on attentional resources involuntarily directed to those narratives. However, all of these possible explanations for the cognitive factors merit further examination.

In our study, valence was not treated as an independent variable. It is worth noting that there is an extensive literature that examines how valence, as one dimension of emotionality, differentially affects memory performance, using positive and negative items (Lavoie and O’Connor, 2013; Hourihan et al., 2017; Bowen et al., 2018; Megalakaki et al., 2019). For instance, Bayer and Schacht (2014) compared the emotional effects of words, pictures, and facial expressions based on ratings and brain imaging data. They found that that while emotional effects were observed on all types of stimuli, differences in valences were reflected in modulations of selected brain waves as well as in ratings. Our ANEW analysis on the narratives provided hints of a role of valence in distinguishing between emotional and neutral transcripts. Further pursuit of valence differences on words and their relation to verbal memory is a topic for further investigation.

One potential limitation of this study is the sample size. The study needs to be replicated with a larger number of participants to provide stronger support for the generalizability of results. The findings of this study have practical implications for educational settings. Incorporating an emotional text to educational materials is more likely to lead to better learning outcomes by facilitating retention of knowledge. Such materials could potentially promote effective learning as students tend to retain verbal material when tinged with emotional nuance. The current findings are also potentially useful for writers who want to engage and influence their readers. Writing strategies that integrate emotionally charged elements will improve reader’s retention, potentially influencing their opinions.

## Conclusion

To conclude, the findings of the present study argue in favor of the existence of verbatim memory for written language and provide further evidence that verbatim memory is boosted by emotional nuance. Taken together, current findings provide support for the episodic theory and the constructionist account of language and contribute to existing knowledge on memory by establishing a link between emotion and verbatim memory.

## Data Availability Statement

The datasets generated for this study are available on request to the corresponding author.

## Ethics Statement

The studies involving human participants were reviewed and approved by the NYU IRB/University Committee on Activities Involving Human Subjects. The patients/participants provided their written informed consent to participate in this study.

## Author Contributions

All authors contributed to the design of the study, participated in the development of stimulus materials, and approved the final version of the manuscript before submission. YK performed data collection, data analysis, and interpretation under the supervision of DS and JS. YK drafted the manuscript. DS and JS provided critical revisions and comments.

## Conflict of Interest

The authors declare that the research was conducted in the absence of any commercial or financial relationships that could be construed as a potential conflict of interest.
