# Supplementary material for: Emotional Nuance Enhances Verbatim Retention of Written Materials
Source: Front Psychol. 2021 Jun 14;12:519729. doi: 10.3389/fpsyg.2021.519729 (PMC8236806; doi:10.3389/fpsyg.2021.519729)
Supplement: Supplementary file 1 [file Data_Sheet_1.docx]

Appendices

# List of emotional narratives used in the study

Emotional narrative #1

…which is like the most exciting thing cause it’s like he’s gonna be there. Us uh – ultimately that’s like all that like matters is like is that he’s there and anybody else who doesn’t like anything can leave. But like I’m so excited because I don’t know. We get to like do all of this stuff. And and I’ve sort of made up for last time like getting on the Pinterest and and looking up all this stuff. And like you know we’re about to write vows. That’s kind of terrifying. Cause I I feel like his vows are gonna be better than my vows. His vows are gonna be so much better than mine. And I’m gonna be like I love you, you know, cause I like freeze up. I freeze up with – I can be angry really easily. But like showing like love I get like I get nervous cause I’m not that kind of person. Like on you know whatever… but yea I’m just excited. And then the engagement was amaze – see he’s just like – it makes me like - I’m so happy, but he’s so amazing. He’s so amazing. And I just like can never, I can never meet up to his level because like even our engagement.

Emotional narrative #2

So last week I got cast in a new NYMF show at the New York musical theatre festival. And the show is called Claudio Quest which is like about Super Mario brothers except it’s a spin-off. So Claudio would be Mario and Luis is a character who would be Luigi. And then there’s two princesses. And luckily I got the younger sister princess who is- she’s bored and spunky. So I got to go in and wear converse and a romper instead of a pretty princess dress. And when I went to the audition I went in and I took a crown that I bought from party city that was $2. And I went into the room and everyone loved my crown. And I started to sing a song. And I used the crown in my song which was normally a love song if you would sing it normally in an audition, but actually sang- it’s sort of a love song about letting someone go. But instead of singing it about a person I sang it about my crown because I didn’t want to be a princess…

Emotional narrative #3

…my background and career choice, which is an actor uh... and I let’s see I started doing study at UCB like five years ago probably, which seems uh like to me like a very long time ago. but I understand that in the scheme of life it’s not that long at all. but it feels long to me. Um...I think I always probably wanted to be an actor. um like specifically a dramatic actor. I was like I’m never gonna do comedy, I think it’s really stupid. and like people who do it are like dumb and bad. but uh here I am. but I um was not good at dramatic acting. like in high school I would audition for the plays and uh always get either no role or just like the person in the background. Where if you like squinted you could like kind of see my hand waving. Um… so then I found...I went to Fordham university for college and I found…that comedy is very fun and also hard. and it’s not filled with just a bunch of uh idiots that do it. or I guess we all are idiots I don’t know.

Emotional narrative #4

…I’ve been an RA which has been a lot of fun. Um I really enjoyed it. One strange experience I had when I was being an R…board like right outside the desk. And I couldn’t see what they were looking at from my perspective but I just assumed that they were looking at a flyer for the program that night that was called “Chocolate Sex”. So I was like hey guys, hey, hey, hey friends. Do you like that flyer for “Chocolate Sex”? How do you like it? You like that flyer for “Chocolate Sex”? Do you like it? Do you like that flyer? Do you like it? You like it. And they just looked at me. so I, I got out from behind the desk and I saw that they were looking at a flyer that said “Becoming Harriet Tubman”. And there was just a pause. And it was a strange moment for all of us. So then I went back behind the desk and I just pretended that it never happened. Never ever happened.

Emotional narrative #5

…and it’s really exciting...um...you know I actually have been dodging danger, danger lately. today, was my second chance of dodging danger because um last night i was supposed to take amtrak here and what happened, there was a derailment. I was, you know, couldn’t take amtrak and now I walk into my first day at UCB and there is shooting right outside. so I guess I’m, you know, on the cusp of getting hurt every five seconds. so I never know what’s gonna happen. You don’t wanna be around me because you never know what’s gonna happen. and um but I’m really excited to be here and really excited to start off. Obviously starting off with a bang no pun intended. but it’s gonna be a great one and it’s gonna be really fun. Um I have an awesome family back at home in Pennsylvania, in Philadelphia. obviously you can tell by my uh Pennsylvania Dutch accent...um…you know they’re really great. My sister is a little star, little pageant girl, dancer. She’s been on Dance Moms. You know, she’s, she’s a superstar.

Emotional narrative #6

…the Lenox store wasn’t on the MapQuest directions so my dad was like “Oh I have this map. We’ll just follow this map to where we have to go.” Um and then we got lost for 4 hours in Lancaster. It was horrible. And then we get to this intersection in the middle of nowhere, we finally see another car. and my moms like flag them down, ask them which way the turnpike is. And they point in the opposite direction that we’d been going. And my mom freaked out. Flipped, oh my god. Screamed at my dad. She still gets angry when she talks about this. We like pull up to a gas station. She like throws him out of the car. She’s like “you. Go with him. Write down the directions” and like throws a pen and a piece of paper at me. and we go and I write down. And we’re just sitting in the car fuming the whole way home. And when the Mets game started they were playing Philadelphia. And we had to listen to it on the Philadelphia radio station which I was pissed about because I hate the Philly’s. and all I wanted was to be home by the time the game started and we weren’t. It was the worst car trip ever. And I’m still angry about it.

Emotional narrative #7

…wonderful. And I’m kind of relaxed but I’m also kind of stressed. And he says like sit down, and so we sit down. And I’m like, I’m like antsy. I’m like okay we got to get to where we got to get to. And he’s like okay let’s let’s get to the dinner. And I’m like great. And so I get up and I’m like you know – go back to like – walking really fast or whatever. And he’s – he stands up and he’s like wait wait wait wait wait. And I’m like, what? What do you want? I have done everything that you’ve wanted. And I walk up to him and he goes down on a knee. And I don’t even know what he said. He like, I think he like mumbled something cause he was so excited. Cause he was acting so strange the entire time. He was acting so strange. He mumbled cause he was excited and I was like – and I was, I literally, I was like what. What’s happening? What’s happening? And so then I did my cry. And like anybody who knows me, I do like the ugly cry. It’s not like, I’m adorable, it’s like, it’s like I’m a goblin. A goblin cry. And so he was – it was really – but it was really great. It was really great.

Emotional narrative #8

…got up really fast and I was like oh yeah I’m fine. And my dad was like, “are you okay?” And I was like, “yeah I’m fine.” And then after about 5 minutes my hand blew up and it was completely black. And I was like “uh that hurts- it doesn’t feel very good.” And then uh you know later in the day it really started to blow up and they kept calling me pillow hand. And so I was just like pillow hand for the weekend. And then my dad was like you’ll be fine just shake it off. So I was like okay. And then when I got back to my house with my mom she was like, “Oh my god what happened to your hand?” And I was like I don’t know I just like fell off Parcheesi and I kind of landed on it. She’s like, “we’re going to the hospital right now.” So we went to the hospital. And they were like, “oh yea you broke your metatarsals across your whole hand.” And my mom was so mad at my dad because he was like ‘you’re fine’ and when really I broke my entire hand. And then I had to wear this brace on it for a month. And so I just had to have my hand like this always. And that was in 4th grade. And that’s when I guess…

Emotional narrative #9

…told them I was like ‘oh you know it’s my...whatever my birthday is coming up, but I don’t wanna do anything.’ And they’re like, ‘Yeah, yeah, of course yeah you don’t, we won’t do anything. We’ll just kind of um...we’ll like maybe like get drinks or something.’ And I was like ‘Great. Perfect. It’s all I want. Great.’ And so...um...on my birthday I went to meet them in the city at this place for drinks. and lo and behold of course I walk in and it was like everyone like surprised me. And I was so mad, which is like terrible. Like my friends went through all the effort of like finding all my friends. They invited some family from out of town and I was like ‘what is this!’. I was like ‘I don’t wanna be here.’ And I was so mad for like the first hour and a half. um and I’m just like a terrible person that’s what I realized. Realizing telling these stories. Um but later on in the night I was like ‘Okay. This is fine. And we went out to dinner. And anytime I can get any kind of food if it’s free or whatever I will be a million times happier than I am. So it ended up being really good.

Emotional narrative # 10

…special to me. I’ve never had a real dog. Um so that was my 12th birthday. And let’s see here. 21st birthday is coming up. That’ll be fun. I’ll be here. Um my parents are visiting. I think we’re gonna see the Lion King. I’m excited about that. I’ve never seen the Lion King, and every loves the Lion King, so that’ll be good. I actually just recently watched a video of a sing-off between the casts of the Lion King and Aladdin. They had a sing-off in an airport, weird. And I wish I was there, but I watched it through the magic of the Internet. And um I’m excited to see that…oh I use to do musicals. I forgot about that time in my life. Uh weird I did musicals for a long time. Yea I had a main part once that was cool. And the director actually threatened to uncast me because I was so bad at dancing. He threatened to literally like cut me out of the cast because I could not dance.

Emotional narrative #11

so I went to college and it was like, woohoo. And it was just a whole new experience for me. so my very first month in my first semester of college, I got pretty pretty drunk. and um I fell off my bunk bed. broke my foot in three places. Horrible. and you know I don’t really remember any of it because you know, obviously there’s stuff going on there. and I didn’t know anything about New York, didn’t know anything about the city. you know just kind of ran out into my dorm and was like ‘Oh my god!’ My foot’s broken, freaking out. and someone was like you gotta go to the hospital. And I was like yeah obviously, I broke my foot. so I run to the corner and hail a cab, and say take me to the nearest hospital. happens to be Bellevue, which is, you know, a little scary there. you know, was there, was there for a while. saw some shit. got a little, freaked out. you know, ended up calling my dad who works in pharmaceuticals. and he was just, you know, get out of there. they’re gonna cut your foot off. so…

Emotional narrative #12

…start talking about comic books. And they’re like, really? Really? You think that Captain Marvel…And I’m like you’ve never even read a comic book you just read Wikipedia. And when I say boys I actually mean my brother. My brother does this all the time. Every time we see a super hero movie we leave and he’ll just be like, “Uh like that they’re gonna do this next, and then, you know. They could really do this character.” And then I’m like you literally have never even read it. You just read what happened in the comic book on Wikipedia. And now you’re gonna act like you’re this big expert about it. Then I’m like you know I actually read this. And he’s like “did you? I don’t know who that was..” and I’m like ugh, no stop it. Stop it. So then me and my little brother when we saw the most recent Avengers together without my older brother who always does that. And when we left my little brother looks at me. he goes “you know it was nice seeing this with you without Ralph. Uh cause Ralph is really annoying when we go see these movies cause he just wants to tell us about it all the time.” And I’m like, “I feel so validated right now by you saying this to me.” Um I also felt really validated by my little brother’s college essay because he wrote in it,

# List of neutral narratives used in the study

Neutral narrative #1

…a lot of equipment that I help manage. Uh I actually just got a job here as an equipment manager, so I take care of all the cameras. I make sure they’re in good shape. As well as our lighting equipment, and our audio equipment. Um it’s been great learning about how different equipment works. And I really feel like I’ve learned a lot and grown a lot in my job. Um, and sometimes on the side I work with some of my friends here and we make little films, uh, on our off time. Uh it’s been really great to be part of a community that has such creative people here. It’s been a lot of fun… Right now I’m working on a series with a friend, uh, about break ups. And we’re exploring – and it’s a comedic series - so we’re exploring different breaks ups in different neighborhoods of New York. And she plays all the characters. Um, it’s been really really great to film that…

Neutral narrative #2

I’m going to a bachelorette party in Florida. My girlfriend’s getting married. She um is marrying someone she’s been with for about five years. and we all rented a big house on the water. There are about twelve bedrooms. Um...and it should be a blast. Uh we’re just gonna hang out in the beach and get tan, and drink lots of alcohol and um...make each other laugh and probably take some selfies and um… some, you know, bikini photographs and such and then at night, we plan on going out. uh I’m not really sure to where. but one of the girls in the bridal party, she uh is from Fort Lauderdale and she plans to show us around. I kinda do wish that it was happening in Miami because there’s more things to do there. and…

Neutral narrative #3

…in uh twenty-fourteen I turned uh 24 and uh I recently got together with my kindergarten best friend and she said, why don’t you come out to New Brunswick with all of me and my friends and we’ll have a good time. We’ll go to brother Jimmy’s, which is a bar in New Brunswick and I invited my friend from high school, Ariel, with us. and we got to the bar and one of her friends was buying us uh pickle-backs. There’re uh these shots of pickle juice and whisky. and you do the whisky and then you do the pickle juice and there’s no bite to the whisky whatsoever. so you don’t even taste the whisky. So one of her friends was buying me and my friend Ariel those shots all night.

Neutral narrative #4

…definitely a good, a good waste of time in the sense of a party but for young girls it took a lot more time than the expert. And I remember vividly that all the girls in this moment would watch the owner of the store in amazement to see how she had done something so quickly that we had just spent an hour and a half to two hours doing. And then of course everyone would eat cake afterwards wearing their new necklaces. And get to go home…point on we would go back to my house and do a lot of face paint. And also um eat ice cream Hoodsie cups which I’m not sure if that’s just a regional thing. But where I’m from that’s definitely a staple at all birthday parties – is a small ice cream cup with vanilla or chocolate, they come together half and half. And you eat it with a small wooden flat spoon stick. And girls always leave with…

Neutral narrative #5

…uh 8th avenue was completely blocked off...um.. there was a shooting I guess this morning. Um...somebody uh a man wielding a hammer uh was shot by police. um I told a comedy friend of mine and his first instinct was ‘Oh my god, they shot Thor.’ I know that’s probably not a funny subject that we should be talking about, but um it happened. and it’s something that I’ve seen uh more than once on the streets of New York. uh whole avenues blocked off um due to shootings and people uh gathered around, fascinated to know what happened... um…Uh you walk around New York and all kinds of crazy things happen, everything is so on top of- on top of each other um... it’s a great city, though. Let’s see uh we’ve got the warm weather now. And I have been spending my days uh at the New York public library and in Bryant park, which is…

Neutral narrative #6

…chalkboard when I was younger that I would hide behind and put puppets out on the side because it was my make-shift kind of puppet- puppet stage that I would use. Um so throughout my life I would get involved in the school plays. I would um like write little like dance numbers with my girlfriends. Um but I never really took any acting classes or anything for a while because um my parents were more- they encouraged me creatively but it was more like- where I grew up it was more likely for people to play sports and get involved in sports there. The arts weren’t as heavily influenced in the school systems as sports were as I feel like is a thing everywhere. Um so as I grew um I had found out about uh the UCB which was for comedy. And I always knew when it came to performing and entertaining people I was

Neutral narrative #7

and we just bought a trip down to Turks and Caicos for the end of June for our um annual sister scuba diving adventure. Um...I’m hoping that when we get there someone will give us a brush up course because when you’re that far away from that world for so long, you kinda remem, like you don’t remember the safety precaution that you have to take. So hopefully someone will show us exactly what we need to do in a pool or something before we get out into the water. Uh we’re staying at a really nice resort that’s right on the beach. And uh the pictures look really beautiful. I’m particularly excited about going on the nights dive because there’s lots of animals there …that’s one thing that we will see on the night dive. Um and then after that, they let us use those little glow lights so that we can see our way around. And also

Neutral narrative #8

and my grandma go on these road trips in our RV. It’s a mini Winnebago. And uh…they’ve gone everywhere in it. I’ve only gone uh to, uh, the furthest I’ve gone is California. But they’ve gone to Washington, they’ve gone to Oregon, they’ve gone uh to all of the states that border Canada. Um…I think, I think I’ve been to North and South Dakota. Uh in fact I’m, I’m sure I’ve been to North and South Dakota, but not so close to the Canada border that they’ve been to. Um uh they they do road trips all the time. And sometimes they’re, they’re gone for like three or four weeks. Um and that’s why I don’t go with them anymore cause I have a career. Um but I had a lot of fun going to California with them the last time I went. Um we drove across the country to California

Neutral narrative #9

whole family. And the best part of the wedding was definitely the appetizers. There were uh mini, mini sliders, mini hot – uh hamburgers, hot dogs, shooter oysters. Lots of drinks. Um it was very fun. Also they had a giant cheese display and and table that smelled terrible but definitely was really yummy. And…beautiful toasts – they did kind of a bring your own dessert things. So they had five or so friends who were really good bakers. And they all baked cakes or cupcakes and cookies. And then there was a mini competition for the best wedding cake. One of them was red velvet. I remember that was the first time I’d ever had red velvet cake. And um one of the cakes was a carrot cake and my aunt actually decorated that cake. And she plucked a flower from the hotel and put it on the cake. And then later we

Neutral narrative #10

and anything that we can get our hands on, onto surfaces. Our apartment looks like just a complete mess. um and the cat still insists on finding ways of jump up onto things. So um it’s only getting worse. And I’m gonna take her in today and hopefully they can do something about it. But these little cats are so hard to probe and poke, and x-ray, and figure out what’s wrong with them. Um I’m really(also intensifier) amazed, I’m amazed when I see these vets dealing with dozens of different species on a daily basis. I mean it’s mind-boggling. I think it’s hard enough being a doctor with one species of animal. um but uh we do have the most amazing vet who I would recommend to anybody uh...Dr. Erickson at Blue Pearl. And uh I really have faith that If anybody can figure out what’s wrong with her, it’s him. And uh we can uh get her back to her old happy tumbling self or as much as possible in the next, uh well, give her the next uh couple years I hope. Let her ride it out happily, pain-free.

Neutral narrative #11

Uh right now is graduation season. And I’ve been seeing a lot of my friends’ little sisters and little brothers graduating from high school. And also friends graduating from college. And it’s brought back memories from when I graduated. I graduated high school almost 10 years ago. My, uh, 10 year reunion is coming up…But thinking back about my own graduation, it was a really great day. I mean, my best friends were there. I just remember being really happy. Feeling very accomplished about what I had done. And um since I like filmmaking I brought a camera with me to graduation and my friend and I were able to film our experience throughout the whole thing. We hid the camera in our graduation gowns. …day as well. Um I met my husband in college, so we were able to graduate together. And of course all my family was there. And it’s one of those days where you just feel like you achieved something really big. And

Neutral narrative #12

I have uh family in the mid- a lot of family in the Midwest where my mother’s from. She’s from Detroit. And I- we go out there visit a lot. And ever since I was little I really loved going out to the Midwest. Um it has the tendency to be boring at times just cause where they live there’s a lot of cornfields. Um but we’d always as a family go to the state fair. The Indiana state fair. And that was always a lot of fun just as someone who grew up on the East coast, and in New Jersey we don’t have, that I know of, I’m sure we do have state fairs. But they’re not as big of a to-do where I’m from. So it was always fun to go as a family. All the girl cousins would go. And we’d go see the pigs, and the sheep, and like the cakes that people would make and decorate. And at the Indiana state fair they’ll fry anything for you. So we all would go uh and just I don’t know just find the most

# Topics covered in narratives

| emotional narrative | set | topic | neutral narrative | set | topic |
| --- | --- | --- | --- | --- | --- |
| #1 | A | wedding vow | #1 | A | career (theatre) |
| #2 | A | career (actress) | #2 | A | bachelorette party |
| #3 | A | career (actress) | #3 | A | birthday party |
| #4 | A | career (theatre) | #4 | A | birthday party |
| #5 | A | daily life, family | #5 | A | daily life |
| #6 | A | family trip | #6 | A | career (actress) |
| #7 | B | marriage proposal | #7 | B | family trip |
| #8 | B | family | #8 | B | family trip |
| #9 | B | birthday party | #9 | B | wedding |
| #10 | B | birthday, theatre | #10 | B | pet |
| #11 | B | college life | #11 | B | graduation |
| #12 | B | family | #12 | B | family |

# Video ratings of emotional expressiveness and engagement in narratives

| **emotional narrative** | **measure** | **rating** |
| --- | --- | --- |
| #1 | emotional expressiveness | 6.2 |
|  | engagement | 5.6 |
| #2 | emotional expressiveness | 6.4 |
|  | engagement | 5.6 |
| #3 | emotional expressiveness | 5 |
|  | engagement | 5.6 |
| #4 | emotional expressiveness | 6.6 |
|  | engagement | 6 |
| #5 | emotional expressiveness | 5.6 |
|  | engagement | 6 |
| #6 | emotional expressiveness | 6.8 |
|  | engagement | 6 |
| #7 | emotional expressiveness | 6.2 |
|  | engagement | 5.8 |
| #8 | emotional expressiveness | 6.4 |
|  | engagement | 6 |
| #9 | emotional expressiveness | 6.6 |
|  | engagement | 6.2 |
| #10 | emotional expressiveness | 6.6 |
|  | engagement | 5.6 |
| #11 | emotional expressiveness | 6 |
|  | engagement | 6 |
| #12 | emotional expressiveness | 6.8 |
|  | engagement | 6.2 |

| **neutral narrative** | **measure** | **rating** |
| --- | --- | --- |
| #1 | emotional expressiveness | 1.6 |
|  | engagement | 2.4 |
| #2 | emotional expressiveness | 2 |
|  | engagement | 2.8 |
| #3 | emotional expressiveness | 1.6 |
|  | engagement | 2.4 |
| #4 | emotional expressiveness | 2.4 |
|  | engagement | 3.2 |
| #5 | emotional expressiveness | 2 |
|  | engagement | 2.8 |
| #6 | emotional expressiveness | 2.2 |
|  | engagement | 3 |
| #7 | emotional expressiveness | 1.6 |
|  | engagement | 2.8 |
| #8 | emotional expressiveness | 2.2 |
|  | engagement | 2.8 |
| #9 | emotional expressiveness | 2.4 |
|  | engagement | 2.4 |
| #10 | emotional expressiveness | 2 |
|  | engagement | 2.8 |
| #11 | emotional expressiveness | 2 |
|  | engagement | 2.6 |
| #12 | emotional expressiveness | 2.4 |
|  | engagement | 2.8 |

# List of emotional excerpts

1. which is an actor uh... and I let’s see I started
2. seems uh like to me like a very long time ago. but I understand that
3. I think it’s really stupid. and like people who do it are like dumb
4. bad. but uh here I am. but I um was not good at
5. just like the person in the background. Where if you like
6. …and it’s really exciting...um...you know I actually have been
7. I never know what’s gonna happen. You don’t wanna be around me
8. to be here and really excited to start off. Obviously starting off with
9. but it’s gonna be a great one and it’s gonna be really fun.
10. um...you know they’re really great. My sister is a little star, little
11. my dad was like “Oh I have this map. We’ll just follow this map
12. the middle of nowhere, we finally see another car. and my moms like
13. my dad. She still gets angry when she talks about this.
14. me. and we go and I write down. And we’re just sitting in the car
15. all I wanted was to be home by the time the game started and we
16. So last week I got cast in a new ... show at the
17. And luckily I got the younger sister princess who is- she’s bored
18. And when I went to the audition I went in and I took
19. my song which was normally a love song if you would sing it normally in
20. it’s sort of a love song about letting someone go. But instead of
21. that’s like all that like matters is like is that he’s there and
22. know. We get to like do all of this stuff. And and I’ve sort of
23. can be angry really easily. But like showing like love I get like I get
24. kind of person. Like on you know whatever… but yea I’m just
25. amazing. He’s so amazing. And I just like can never, I can
26. really enjoyed it. One strange experience I had when I was being
27. like right outside the desk. And I couldn’t see what they were looking at from
28. I was like hey guys, hey, hey, hey friends. Do you like
29. Do you like it? You like it. And they just looked at me.
30. moment for all of us. So then I went back behind the desk
31. I don’t wanna do anything.’ And they’re like, ‘Yeah, yeah, of course
32. something.’ And I was like ‘Great. Perfect. It’s all I want.
33. so mad, which is like terrible. Like my friends went
34. so mad for like the first hour and a half. Um
35. later on in the night I was like ‘Okay. This is fine.
36. for me. so my very first month in my first semester of
37. you know I don’t really remember any of it because you know
38. didn’t know anything about the city. you know just kind of
39. you know, was there, was there for a while. saw some
40. and he was just, you know, get out of there. they’re gonna
41. boys I actually mean my brother. My brother does this all the time.
42. I’m like you literally have never even read it. You just read what
43. about it. Then I’m like you know I actually read this.
44. So then me and my little brother when we saw the most recent
45. when we go see these movies cause he just wants to tell us
46. okay?” And I was like, “yeah I’m fine.” And then after about
47. And then uh you know later in the day it really started
48. when I got back to my house with my mom she was like,
49. so mad at my dad because he was like ‘you’re fine’
50. just had to have my hand like this always. And that was in
51. he’s like okay let’s let’s get to the dinner. And I’m like great.
52. like, what? What do you want? I have done everything that you’ve
53. I don’t even know what he said. He like, I think he
54. was excited and I was like – and I was, I literally,
55. it was really – but it was really great. It was really great.
56. special to me. I’ve never had a real dog. Um so
57. is coming up. That’ll be fun. I’ll be here. Um
58. so that’ll be good. I actually just recently watched a video
59. And I wish I was there, but I watched it through the
60. And um I’m excited to see that…oh I use to do

# List of neutral excerpts

1. um I told a comedy friend of mine and his first instinct was
2. know that’s probably not a funny subject that we should be talking about
3. and it’s something that I’ve seen uh more than once on the
4. and all kinds of crazy things happen, everything is so
5. got the warm weather now. And I have been spending my days uh
6. getting married. She um is marrying someone she’s been with for about
7. make each other laugh and probably take some selfies and
8. such and then at night, we plan on going out.
9. I’m not really sure to where. but one of the girls in the
10. show us around. I kinda do wish that it was happening
11. that I help manage. Uh I actually just got a job here
12. like I’ve learned a lot and grown a lot in my job.
13. the side I work with some of my friends here and we make
14. our off time. Uh it’s been really great to be part of a
15. the characters. Um, it’s been really really great to film that
16. so throughout my life I would get involved in the school
17. like dance numbers with my girlfriends. Um but I never really took
18. it was more like- where I grew up it was more likely
19. I feel like is a thing everywhere. Um so as I
20. for comedy. And I always knew when it came to
21. best friend and she said, why don’t you come out to
22. me and my friends and we’ll have a good time.
23. and I invited my friend from high school, ..., with us.
24. we got to the bar and one of her friends
25. So one of her friends was buying me and my friend
26. party but for young girls it took a lot more time than
27. we had just spent an hour and a half to two hours doing.
28. we would go back to my house and do a lot
29. just a regional thing. But where I’m from that’s definitely
30. come together half and half. And you eat it with a
31. anything that we can get our hands on, onto surfaces. Our apartment
32. things. So um it’s only getting worse. And I’m gonna take her
33. figure out what’s wrong with them. Um I’m really amazed,
34. what’s wrong with her, it’s him. And uh we can uh
35. as much as possible in the next,  uh well, give her the next
36. Um...I’m hoping that when we get there someone will give us a
37. far away from that world for so long, you kinda remem, like you don’t
38. you have to take. So hopefully someone will show us
39. uh the pictures look really beautiful. I’m particularly excited about
40. let us use those little glow lights so that we can see our
41. a lot of my friends’ little sisters and little brothers graduating from high
42. And it’s brought back memories from when I graduated. I
43. a really great day. I mean, my best friends were there.
44. in college, so we were able to graduate together. And
45. family was there. And it’s one of those days where you just feel
46. out there visit a lot. And ever since I was little I
47. to be boring at times just cause where they live there’s a lot of
48. as big of a to-do where I’m from. So it was always fun
49. a family. All the girl cousins would go. And we’d
50. anything for you. So we all would go uh and
51. they’ve gone everywhere in it. I’ve only gone uh to,
52. that they’ve been to. Um uh they they do road
53. the time. And sometimes they’re, they’re gone for like three or
54. Um and that’s why I don’t go with them anymore cause I have a
55. with them the last time I went. Um we drove
56. family. And the best part of the wedding was definitely
57. Lots of drinks. Um it was very fun. Also they
58. things. So they had five or so friends who were
59. I remember that was the first time I’d ever had
60. hotel and put it on the cake. And then later
